# Association of Anti‐Inflammatory Dietary Adherence With Biomarkers and Gut Microbiota Related to Colorectal Cancer Risk: A Retrospective Study

**DOI:** 10.1002/fsn3.71497

**Published:** 2026-02-06

**Authors:** Hantao Wang, Yunjie Shi, Wei Wang, Xu Li

**Affiliations:** ^1^ Department of Colorectal Surgery The First Affiliated Hospital of Naval Medical University Shanghai China

**Keywords:** anti‐inflammatory diet, biomarkers, cancer prevention, colorectal cancer, gut microbiota, inflammation

## Abstract

Colorectal cancer is one of the most common cancers and a primary cause of death. The increased incidence in low‐ and middle‐income nations highlights the need for better prevention. Chronic inflammation, obesity, and gut microbial dysbiosis are major risk factors for CRC, making nutritional interventions attractive. This study aims to examine the association between adherence to an anti‐inflammatory diet and anthropometric, biochemical, inflammatory, molecular, and gut microbiota parameters related to colorectal cancer risk. In this retrospective analysis, anti‐inflammatory diet adherents (*n* = 515) and non‐adherents (*n* = 435) were compared. Hematological, hepatic, inflammatory, tumor, genetic/molecular, and gut microbiota tests were performed, and chi‐square tests were used for categorical outcomes. Multiple regression was used to examine the association between adherence to an anti‐inflammatory diet and the development of colorectal cancer. Multiple logistic regression analysis indicated that anti‐inflammatory diets were associated with improved clinical, biochemical, and microbiome outcomes in patients with CRC. Diet adherence was associated with a lower risk of obesity, central obesity, dyslipidemia, anemia, and leukocytosis after adjusting for age, sex, BMI, smoking, and caloric intake (*β* = −1.90, SE = 0.26, OR = 0.15, 95% CI: 0.09–0.25). Several inflammatory markers, including CRP, IL‐6, CEA, and MMP‐9, decreased markedly (*p* < 0.001). Molecular alterations associated with CRC, including p53 mutation, Ki‐67 overexpression, microsatellite instability, APC mutation, and β‐catenin nuclear expression, were significantly decreased (*p* < 0.001). *Bifidobacterium*, *Lactobacillus*, *Faecalibacterium prausnitzii* , and *Akkermansia muciniphila* were increased, and pathogenic species decreased in the gut (ORs 2.10–2.30; *Fusobacterium nucleatum* , *Clostridium difficile* , pathogenic *Escherichia coli* ; ORs 0.16–0.18). Anti‐inflammatory diets significantly improve metabolic, inflammatory, tumor‐related, and microbiome profiles in patients with CRC. Adherence to an anti‐inflammatory dietary pattern is significantly associated with improved metabolic, inflammatory, molecular, and gut microbiota profiles, all of which are linked to colorectal cancer risk. These findings support anti‐inflammatory dietary strategies as cost‐effective and non‐invasive approaches for colorectal cancer prevention and adjunctive management.

## Graphical abstract

This retrospective study evaluates whether greater adherence to an anti‐inflammatory diet is associated with biomarkers and gut microbiota profiles linked to reduced colorectal cancer risk. The study seeks to clarify how dietary patterns influence inflammation and gut microbiota, providing insights to guide effective prevention strategies for individuals at risk of colorectal cancer.

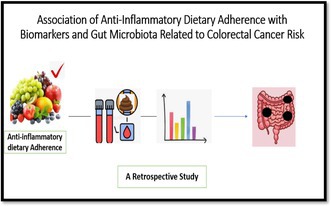

## 1 Introduction

Colorectal cancer (CRC) is a leading cause of cancer morbidity and mortality worldwide. According to the International Agency for Research on Cancer (IARC), CRC is the third most commonly diagnosed cancer and the second largest cause of cancer deaths worldwide, accounting for 10% of new cancer cases and 9% of cancer‐related fatalities (Xi and Xu 2021). Over 930,000 fatalities and 1.9 million new cases were reported in 2020, and aging populations and global adoption of Western diets and lifestyles are likely to increase the impact (Olfatifar et al. 2025). CRC is increasing quickly in many low‐ and middle‐income countries. Asia had half of the global CRC cases and fatalities in 2022, 966,400 new cases and 462,300 deaths (Khan and Lengyel 2023). In 2022, China had 517,100 new colorectal cancer cases, making it one of the most common malignancies in the world, with a high death rate (Qu et al. 2022). These trends highlight the global public health burden of CRC and the urgent need to reduce it by addressing modifiable risk factors including diet, obesity, physical inactivity, and chronic inflammation. The complex interplay of non‐modifiable and modifiable risk variables affects genetic predisposition, environmental exposures, and CRC‐causing biological pathways (Hossain et al. 2022). Age is the strongest non‐modifiable factor, with incidence rising after 50. Due to germline DNA mismatch repair gene mutations, Lynch syndrome and FAAP increase the lifetime CRC risk and are also risk factors (Carr et al. 2018; Padmanabhan et al. 2018; Yang, Li, et al. 2019). CRC risk increases with nitrosamine formation, heme iron‐induced oxidative stress, and high‐temperature cooking, which generates carcinogenic heterocyclic amines. Insufficient whole grains, fruits, vegetables, and dietary fiber limit butyrate synthesis, which protects colonic epithelial integrity and modulates immunological responses. Obesity, central obesity, and physical inactivity increase insulin resistance, hyperinsulinemia, and IGF‐1 signaling, which promote cell proliferation and inhibit apoptosis, thereby increasing CRC risk. Heavy drinking exposes people to acetaldehyde, whereas tobacco use adds colon cancer‐causing mutagenic chemicals (Li et al. 2022; Yang et al. 2025).

Additional medical and biological factors contribute to CRC. Chronic cytokine release, oxidative stress, and epithelial degradation accelerate the adenoma–carcinoma pathway in ulcerative and Crohn's colitis. Type II diabetes and metabolic syndrome also increase CRC risk through low‐grade inflammation (Li et al. 2022; Muro et al. 2024). Loss of butyrate‐producing commensals deprives the colon of anti‐inflammatory and anti‐carcinogenic metabolites, whereas *Fusobacterium nucleatum* or enterotoxigenic enrichment *Bacteroides fragilis* destroys DNA, breaks the epithelial barrier, and affects local immunity. Converging risk factors indicate that CRC etiology is complex and that diet and lifestyle modifications are required to prevent it. Inflammation promotes colorectal cancer malignancy through molecular and cellular pathways. Chronic mucosal inflammation with inflammatory bowel disease increases epithelial injury and regeneration, increasing DNA damage and mutagenesis. Chronic activation of NF‐κB and COX‐2 pathways promotes epithelial proliferation, suppresses apoptosis, promotes angiogenesis, and alters cell invasiveness (Ahmad et al. 2024; Aran et al. 2016; Sohrab et al. 2023). Pro‐inflammatory cytokines and mediators such as IL‐6, TNF‐α, and prostaglandins, exacerbate the tumor‐permissive environment by inducing oxidative stress, impairing DNA repair, and reducing anti‐tumor immunity. *Fusobacterium nucleatum* and enterotoxigenic *B. fragilis* overgrowth in the gut microbiome can produce genotoxic compounds, alter epithelial barrier integrity, and regulate host immunological signaling to promote tumor initiation and progression (Joo et al. 2024). Short‐chain fatty acids that regulate inflammation, maintain mucosal health, and combat cancer become scarcer as butyrate‐producing commensals dwindle. By releasing adipokines and pro‐inflammatory mediators from excess adipose tissue, obesity‐related metabolic inflammation produces chronic low‐grade systemic inflammation. Hyperinsulinemia and IGF signaling promote colonic epithelial cell growth and survival. This pathway shows how chronic inflammation, microbial imbalance, and metabolic dysregulation promote colon cancer (Kasprzak 2023; Khanna et al. 2022).

Pro‐inflammatory diets increase CRC risk, whereas anti‐inflammatory diets, such as the Mediterranean diet and those rich in whole grains, fruits, vegetables, legumes, seafood, and nuts, reduce it. Anti‐inflammatory foods protect in many ways. High fiber consumption increases colonic fermentation and produces short‐chain fatty acids, primarily butyrate, which serve as an energy source for colonocytes and inhibit histone deacetylases, thereby increasing cancer cell death and decreasing pro‐inflammatory gene expression (Shivappa et al. 2017; Sohrab et al. 2023). CRC is the primary cause of cancer‐related morbidity and mortality worldwide, and diet and inflammation contribute to its development. Observational studies show that an anti‐inflammatory diet reduces CRC risk via modulating inflammatory pathways and gut flora. Diet is adjustable and scalable; therefore, observing its association with CRC risk in real‐world populations can inform preventative measures. Thus, this study aims to examine the association between adherence to an anti‐inflammatory diet and anthropometric, biochemical, inflammatory, molecular, and gut microbiota parameters related to colorectal cancer risk.

## 2 Materials and Methods

### 2.1 Ethical Considerations

The study was approved by the Ethics Committee of Shanghai Changle Hospital, China (approval no. SC26111231), where data were retrieved from the hospital's electronic medical record system and supplemented by reviews of physical hospital files. All methods followed the Declaration of Helsinki (2013 revision) ethical norms. Informed consent was waived for this retrospective investigation. Anonymizing records and restricting access to authorized workers protected patient data. This study was conducted as a retrospective observational analysis over 2 years, spanning from November 2022 to January 2025.

### 2.2 Study Participants

This retrospective study examined 1050 electronic medical records of CRC patients, of which 950 met the eligibility criteria. Eligible participants were adults aged ≥ 35 years with a histologically confirmed diagnosis of CRC, documented TNM staging, and sufficiently complete clinical records to allow reliable evaluation. Only patient records that contained sufficient dietary information to calculate the Dietary Inflammatory Index (DII) and relevant laboratory or microbiology results were included in the study. Records were excluded if important clinical, nutritional, or lab data were missing, or if the patient had genetic colon cancer syndromes, other primary cancers, serious diseases, or was on experimental treatments. Laboratory and microbial results were included only if obtained within 3 months of the dietary assessment to ensure data consistency.

### 2.3 Data Collection Tool

All data were extracted retrospectively using a standardized data collection form designed to gather demographic, clinical, dietary, and laboratory information from patient files and electronic medical records. DII was calculated from nutrition clinic notes and dietary counseling records to assess dietary inflammatory index. These ratings divided people into anti‐inflammatory diet adherent and non‐adherent groups. Data from patient files included inflammatory biomarkers (CRP, IL‐6, TNF‐α, IL‐10), CRC‐related biomarkers (CEA, MMP‐9, COX‐2, VEGF, NF‐κB, IGF‐1, p53 mutation, Ki‐67, MSI, BRAF and APC mutations, and β‐catenin expression), hematological measurements (hemoglobin, hematocrit, red cell indices, white blood cell counts, and platelet counts), liver function markers, lipid profiles, and apolipoproteins. Patient medical data included fecal samples for evaluation of the gut microbiota. Samples were collected using standardized sterile collection kits, processed within 24–48 h, and examined using proven microbiological methods according to hospital protocols, where available (patient's record). The relative abundance of *Bifidobacterium*, *Lactobacillus*, *Akkermansia muciniphila* , *Faecalibacterium prausnitzii* , *Roseburia* spp., *F. nucleatum* , pathogenic *Escherichia coli* , and *Clostridium difficile* was reported. Only microbiome data from 3 months after dietary evaluation and clinical care were compared to patient records. For consistency, only laboratory and microbiological data from the same clinical management period and an acceptable retrospective interval were included. Two trained researchers independently reviewed and cross‐verified all extracted data and resolved conflicts to ensure dataset accuracy, completeness, and dependability.

### 2.4 Dietary Assessment and Classification

Dietary data were extracted from nutrition clinic records, dietary counseling notes, and patient food frequency questionnaires documented in medical records. Each participant's DII was computed using approved methods based on pro‐ and anti‐inflammatory substances and foods. Macronutrients, micronutrients (vitamins A, C, D, E, B‐complex, minerals), fiber, and bioactive substances that affect inflammation were essential. Participants with DII scores below the threshold were classified as adherents to an anti‐inflammatory diet, whereas those above the threshold were classified as non‐adherent or pro‐inflammatory. Using a grading system ranging from −8.0 (anti‐inflammatory) to +8.0 (pro‐inflammatory), standardized results were achieved (Hébert et al. 2019).

### 2.5 Statistical Analysis

All statistical analyses were performed using SPSS version 25.0. Continuous variables were expressed as mean ± standard deviation (SD) and compared using independent‐sample *t*‐tests. Categorical variables were presented as frequencies and percentages, and group differences were assessed using Chi‐square tests. Values represent adjusted odds ratios (ORs) with 95% confidence intervals (CIs) derived from multivariate logistic regression analyses, controlling for age, sex, BMI, smoking status, physical activity, and total caloric intake. *β* = standardized regression coefficient; SE = standard error. *p*‐value < 0.05 was considered statistically significant (Steel and Torrie 1981).

## 3 Results and Discussion

### 3.1 Socio‐Demographic Profile

The study found no substantial difference in mean age between anti‐inflammatory diet adherents (58.2 ± 8.5 years) and non‐adherents (59.6 ± 9.1 years; *p* = 0.082). Several socio‐demographic characteristics showed significant variations. Adherents were more likely to be female (53.8% vs. 44.4%, *p* = 0.006), have higher education levels (41.8% vs. 25.0%, *p* < 0.001), and reside in metropolitan areas (67.8% vs. 56.3%, *p* = 0.001). Male, rural, and less educated non‐adherents were more common. Similar marital status distribution between groups (*p* = 0.512). Smoking prevalence was much lower among the anti‐inflammatory diet adherent group (13.8% vs. 30.3%, *p* < 0.001). Although not statistically significant (*p* = 0.098), non‐adherents had a 15.6% family history of colorectal cancer compared to 11.8% (Table 1). These findings support previous research suggesting women, urbanites, and college graduates are more likely to eat plant‐forward, anti‐inflammatory diets (Longworth 2022). Similar socio‐demographic trends have been reported in Chinese cohorts and other populations, where higher education and urbanization are associated with increased intake of fruits, vegetables, and whole grains, whereas lower education and rural residence correlate with greater consumption of refined grains and processed foods (Lv et al. 2011; Yu et al. 2022). Moreover, the lower smoking prevalence among adherents is consistent with evidence that health‐conscious behaviors cluster together, reinforcing the protective association between anti‐inflammatory diets and may help to reduce colorectal cancer risk.

**TABLE 1:** Socio‐demographic profile of participants.

Variable	Anti‐inflammatory diet adherents (*n* = 515)	Non‐adherent (*n* = 435)	*p*
Age (years, mean ± SD)	58.2 ± 8.5	59.6 ± 9.1	0.082
Sex (%)			
Male	238 (46.2%)	242 (55.6%)	0.006*
Female	277 (53.8%)	193 (44.4%)	
Education level (%)			
Primary or less	102 (19.8%)	146 (33.6%)	< 0.001*
Secondary	198 (38.4%)	180 (41.4%)	
Tertiary/university	215 (41.8%)	109 (25.0%)	
Residence (%)			
Urban	349 (67.8%)	245 (56.3%)	0.001*
Rural	166 (32.2%)	190 (43.7%)	
Marital status (%)			
Married	402 (78.1%)	332 (76.3%)	
Single/other	113 (21.9%)	103 (23.7%)	
Smoking (%)	71 (13.8%)	132 (30.3%)	< 0.001*
Family history of CRC (%)	61 (11.8%)	68 (15.6%)	0.098

* Chi‐square test was applied for categorical variables; independent‐sample *t*‐test was for continuous variables. *p* < 0.05 is considered significant.

### 3.2 Dietary Intake Patterns Among Anti‐Inflammatory Diet Adherents vs. Non‐Adherents

In this study, among 950 participants, dietary intake differed markedly across nearly every food group examined. Anti‐Inflammatory adherents reported substantially higher consumption of whole grains and traditional cereals (210 ± 45 vs. 110 ± 35 g/day), legumes and soy (160 ± 38 vs. 70 ± 25 g/day), green‐leaf and cruciferous vegetables (220 ± 55 vs. 110 ± 45 g/day), other vegetables (200 ± 50 vs. 100 ± 40 g/day), total fruit (300 ± 60 vs. 150 ± 55 g/day), berries/vitamin‐C fruits (90 ± 20 vs. 40 ± 15 g/day), nuts and seeds (25 ± 8 vs. 10 ± 5 g/day), fish/omega‐3 seafood (3–4 vs. 1–2 servings/week) and fermented/probiotic foods (5–6 vs. 2–3 servings/week) compared with non‐adherents (all *p* < 0.001). Non‐adherents consumed more refined grains (95 ± 25 vs. 230 ± 60 g/day), processed and red meat (0.5 vs. 4–5 servings/week), processed/fried foods (0.5 vs. 3–4 servings/week), and sugary foods/beverages (1 vs. 5 servings/week) and had higher intakes of animal/palm oils (20 g/day plant‐based vs. 40 g/day animal/palm; *p* < 0.001). Use of allium/anti‐inflammatory spices, herbal teas, and daily dairy/alternatives was greater among adherents (*p* ≤ 0.014) (Table 2). In sum, Anti‐Inflammatory adherents exhibited a plant‐based, fish‐ and fermented‐food‐rich pattern, with lower intake of refined carbohydrates, processed meats, and sugary and fried foods, consistent with a lower dietary inflammatory potential. The dietary pattern of anti‐inflammatory adherents in this Chinese cohort—marked by higher consumption of whole grains, legumes, vegetables, fruits, nuts, fish, and fermented foods, and lower intake of refined grains, processed meats, and sugary fried foods closely mirrors Mediterranean‐style and other anti‐inflammatory diets shown in trials such as PREDIMED to reduce inflammatory biomarkers like hs‐CRP and IL‐6 (Yu et al. 2024). Meta‐analyses using the Dietary Inflammatory Index consistently link pro‐inflammatory diets with up to 40% higher CRC risk, and recent development of China‐specific dietary inflammatory indices (CHINA‐DII) further validates the relevance of these food group contrasts in Chinese populations (Chen et al. 2025). Elevated intake of fermented foods among adherents may provide additional protection through microbiome modulation, production of short‐chain fatty acids, and enhanced mucosal immunity (Mann et al. 2024). These data support the biological possibility that anti‐inflammatory diets may modify the development of colorectal cancer, although biomarker validation and long‐term prospective studies are needed to establish causality.

**TABLE 2:** Food group intake among anti‐inflammatory diet adherents vs. non‐adherents in China.

Food group	Food examples	Anti‐inflammatory diet adherents (*n* = 515)	Non‐adherent (*n* = 435)	*p*
Whole grains & traditional cereals	Brown rice, black rice, millet, sorghum, buckwheat, oats, barley, corn, quinoa, rye, whole wheat noodles, and multigrain bread	210 ± 45 g/day	110 ± 35 g/day	< 0.001
Refined grains (pro‐inflammatory)	White rice, polished noodles, mantou (white steamed bun), fried dough sticks (youtiao), rice cakes, white bread, instant noodles, wonton wrappers, dumpling skins, glutinous rice, and sweet buns	95 ± 25 g/day	230 ± 60 g/day	< 0.001
Legumes & soy products	Soybeans, tofu, soy milk, edamame, mung beans, red beans, black beans, chickpeas, lentils, tempeh, natto, and pea protein foods	160 ± 38 g/day	70 ± 25 g/day	< 0.001
Green leafy & cruciferous Vegetables	Bok choy, Chinese cabbage, spinach, kale, mustard greens, broccoli, cauliflower, rape greens, snow pea shoots, water spinach, and Brussels sprouts	220 ± 55 g/day	110 ± 45 g/day	< 0.001
Other vegetables	Carrot, cucumber, tomato, eggplant, pumpkin, bitter melon, lotus root, daikon radish, zucchini, bell pepper, mushroom, and winter melon	200 ± 50 g/day	100 ± 40 g/day	< 0.001
Allium & anti‐inflammatory vegetables	Garlic, onion, scallion, leek, shallot, chive, ginger, galangal, turmeric root, Chinese celery, and coriander	Frequent use	Occasional use	< 0.001*
Fruits (common)	Apple, pear, orange, banana, peach, watermelon, tangerine, grape, kiwi, persimmon, plum, and pomegranate	300 ± 60 g/day	150 ± 55 g/day	< 0.001
Berries & vitamin C‐rich fruits	Goji berries, hawthorn, strawberry, blueberry, mulberry, bayberry, longan, lychee, rambutan, cherry, and dragon fruit	90 ± 20 g/day	40 ± 15 g/day	< 0.001
Nuts & seeds	Walnut, almond, peanut, sunflower seed, pumpkin seed, sesame seed, flaxseed, cashew, chestnut, hazelnut, and pine nut	25 ± 8 g/day	10 ± 5 g/day	< 0.001
Fish & seafood (omega‐3 rich)	Salmon, mackerel, sardine, anchovy, crucian carp, grass carp, silver carp, shrimp, crab, clam, eel, and squid	3–4 servings/week	1–2 servings/week	< 0.001
Other seafood	Sea cucumber, jellyfish, seaweed, kelp, mussel, oyster, abalone, scallop, cuttlefish, crayfish, and ribbonfish	2 servings/week	< 1 serving/week	< 0.001
Poultry & lean meats	Chicken, duck, goose, turkey, lean pork, rabbit, pigeon, quail, lean beef, lamb (lean cuts), and organ meats (moderate)	2–3 servings/week	5–6 servings/week (red/fatty meats)	< 0.001
Processed & red meats (pro‐inflammatory)	Fatty pork, beef brisket, lamb hotpot cuts, bacon, ham, sausage, luncheon meat, Chinese preserved meats (lap cheong), barbecued pork (char siu), and fried chicken	0.5 serving/week	4–5 servings/week	< 0.001
Fermented & probiotic foods	Pickled cabbage (low‐salt), suan cai, kimchi, natto, miso, fermented tofu, yogurt, kefir, kombucha, fermented soy paste, fermented rice drinks, and tempeh	5–6 servings/week	2–3 servings/week	< 0.001
Herbal teas & beverages	Green tea, oolong tea, Pu'er tea, chrysanthemum tea, goji berry infusion, ginger tea, lotus leaf tea, hawthorn tea, jasmine tea, barley tea, and buckwheat tea	2–3 cups/day	< 1 cup/day	< 0.001
Dairy & alternatives	Milk, yogurt, kefir, soy milk, goat milk, cheese (low intake in China), milk powder, probiotic drinks, almond milk, oat milk, and rice milk	1 serving/day	< 0.5 serving/day	0.014
Oils & fats	Sesame oil, peanut oil, soybean oil, olive oil, canola oil, sunflower oil, flaxseed oil, lard, butter, palm oil, and blended cooking oils	20 g/day (plant‐based oils)	40 g/day (animal/palm oils)	< 0.001
Spices, herbs & condiments	Garlic, ginger, scallion, coriander, cumin, fennel, star anise, cinnamon, Sichuan pepper, sesame oil, turmeric, and chili	Frequent use	Occasional use	0.002*
Processed & fried foods (pro‐inflammatory)	Fried chicken, sweet pastries, potato chips, fried rice, fried noodles, packaged snacks, sausages, bacon, ham, instant ramen, deep‐fried tofu, and sugar‐sweetened beverages	0.5 serving/week	3–4 servings/week	< 0.001
Sugary foods & beverages (pro‐inflammatory)	Bubble tea, fruit juices (sweetened), soda, sweet cakes, mooncakes, egg tarts, candied fruits, ice cream, shaved ice desserts, sweet rice balls, and sweet soy drinks	1 serving/week	5 servings/week	< 0.001

*Note:* Values are expressed as mean ± standard deviation (SD) for continuous variables (g/day or servings/week) and as frequency of use for qualitative variables. Group comparisons were performed using independent‐sample *t*‐tests for continuous variables and Chi‐square tests (*) for categorical variables. A two‐tailed *p* < 0.05 was considered statistically significant.

### 3.3 Body Composition and Clinical Outcomes in Anti‐Inflammatory vs. Non‐Adherent Groups

Table 3 presents the expanded anthropometric characteristics of participants stratified by adherence to an anti‐inflammatory diet. Individuals in the anti‐inflammatory group had significantly lower mean body weight (64.5 ± 7.8 vs. 82.3 ± 10.1 kg, *p* < 0.001) and BMI (22.7 ± 2.2 vs. 29.8 ± 3.3 kg/m^2^, *p* < 0.001) compared with non‐adherents. Central adiposity markers were also markedly reduced in the anti‐inflammatory group including waist circumference (79.6 ± 6.8 vs. 99.2 ± 8.9 cm, *p* < 0.001), waist–hip ratio (0.85 ± 0.04 vs. 0.93 ± 0.05, *p* = 0.009), body fat percentage (22.2% ± 4.0% vs. 34.1% ± 5.7%, *p* < 0.001), visceral fat (76.9 ± 15.9 vs. 135.4 ± 28.2 cm^2^, *p* < 0.001), and subcutaneous fat thickness (14.2 ± 3.1 vs. 25.6 ± 4.8 mm, *p* < 0.001). Extremity circumferences were also smaller among adherents, with lower mid‐upper arm (26.3 ± 2.8 vs. 32.5 ± 3.5 cm, *p* < 0.001), thigh (49.5 ± 4.2 vs. 57.9 ± 5.1 cm, *p* = 0.004), and calf (34.1 ± 2.9 vs. 39.6 ± 3.4 cm, *p* = 0.008) values. The anti‐inflammatory group had considerably larger skeletal muscle mass (28.6 ± 3.4 vs. 24.2 ± 3.1 kg, *p* < 0.001) and lean body mass percentage (75.4% ± 5.6% vs. 65.9% ± 6.1%, *p* < 0.001), indicating a better body composition. Adherents had a slightly lower basal metabolic rate (1452 ± 130 vs. 1638 ± 155 kcal/day, *p* = 0.012) due to lower body mass. Adherents had significantly higher physical activity levels, as reflected by higher IPAQ scores (2650 ± 420 vs. 1480 ± 350; *p* < 0.001). The anti‐inflammatory group had significantly lower prevalence of sarcopenia (6% vs. 19%, *p* < 0.001) and central obesity (18% vs. 67%, *p* < 0.001) in clinical outcomes. These data show that an anti‐inflammatory diet reduces adiposity, increases muscle mass, increases physical activity, and reduces the risk of sarcopenia and central obesity (Shahinfar et al. 2022). Large‐scale prospective studies and meta‐analyses link central and visceral obesity to colorectal cancer more than BMI. To succeed, followers need a low waist circumference, waist–hip ratio, and visceral fat (Atakan et al. 2021; Kolb 2022). Adherents had significantly higher IPAQ scores, consistent with evidence that exercise lowers inflammation, improves insulin sensitivity, preserves lean mass, and reduces visceral fat, reducing CRC risk (Li et al. 2023). Our cross‐sectional findings are supported by trial data that Mediterranean‐style, anti‐inflammatory diets and physical activity minimize visceral fat and lean mass loss. Reducing visceral adiposity can improve intestinal homeostasis by reducing systemic inflammation, circulating cytokines (e.g., IL‐6, TNF‐α), and pro‐tumorigenic adipokines, whereas also encouraging a gut microbiome rich in short‐chain fatty acids (Ma et al. 2024). Overall, individuals who adhere to an anti‐inflammatory diet have lower adiposity, greater lean mass, higher physical activity, and lower rates of sarcopenia and central obesity. Given their substantial associations with CRC risk and outcomes, an anti‐inflammatory diet and increased physical activity may influence CRC development.

**TABLE 3:** Anthropometric parameters in anti‐inflammatory vs. non‐adherent groups.

Parameter (*N* = 950)	Anti‐inflammatory diet adherents (*n* = 515)	Non‐adherent (*n* = 435)	*p*
Body weight (kg)	64.5 ± 7.8	82.3 ± 10.1	< 0.001
BMI (kg/m^2^)	22.7 ± 2.2	29.8 ± 3.3	< 0.001
Waist circumference (cm)	79.6 ± 6.8	99.2 ± 8.9	< 0.001
Hip circumference (cm)	93.8 ± 6.3	107.6 ± 8.1	0.002
Waist–hip ratio	0.85 ± 0.04	0.93 ± 0.05	0.009
Body fat (%)	22.2 ± 4.0	34.1 ± 5.7	< 0.001
Visceral fat (cm^2^)	76.9 ± 15.9	135.4 ± 28.2	< 0.001
Subcutaneous fat thickness (mm)	14.2 ± 3.1	25.6 ± 4.8	< 0.001
Mid‐upper arm circumference (cm)	26.3 ± 2.8	32.5 ± 3.5	< 0.001
Thigh circumference (cm)	49.5 ± 4.2	57.9 ± 5.1	0.004
Calf circumference (cm)	34.1 ± 2.9	39.6 ± 3.4	0.008
Skeletal muscle mass (kg)	28.6 ± 3.4	24.2 ± 3.1	< 0.001
Lean body mass (%)	75.4 ± 5.6	65.9 ± 6.1	< 0.001
Basal metabolic rate (kcal/day)	1452 ± 130	1638 ± 155	0.012
Physical activity score (IPAQ)	2650 ± 420	1480 ± 350	< 0.001
Sarcopenia prevalence (%)	6%	19%	< 0.001*
Central obesity prevalence (%)	18%	67%	< 0.001*

*Note:* Values are presented as mean ± standard deviation (SD) for continuous variables and as percentages for categorical variables. Independent‐sample *t*‐tests were used to compare continuous variables, and Chi‐square tests (*) were applied for categorical outcomes.

### 3.4 Lipid Profile Differences Between Anti‐Inflammatory Diet Adherents and Non‐Adherents

**FIGURE 1:**
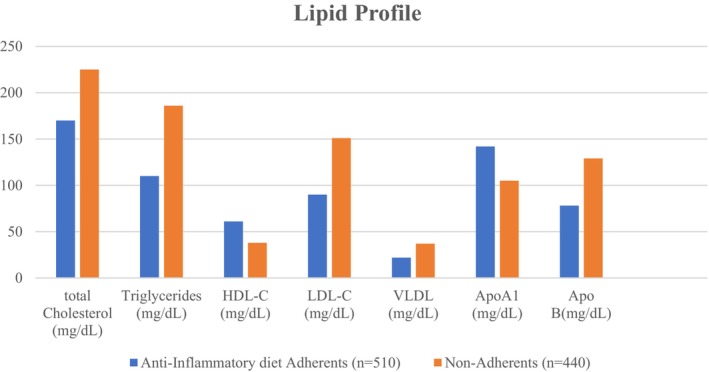
Lipid profile differences between anti‐inflammatory diet adherents and non‐adherents.

Figure 1 summarizes participants' lipid profiles by dietary adherence. Individuals following an anti‐inflammatory diet demonstrated significantly more favorable lipid parameters compared to non‐adherents. Total cholesterol levels were markedly lower in adherents (170 ± 24 vs. 225 ± 38 mg/dL, *p* < 0.001), along with reduced triglycerides (110 ± 30 vs. 186 ± 47 mg/dL, *p* < 0.001) and LDL‐C concentrations (90 ± 20 vs. 151 ± 34 mg/dL, *p* < 0.001). VLDL was also significantly decreased (22 ± 6 vs. 37 ± 11 mg/dL, *p* = 0.003). In contrast, HDL‐C, the protective fraction, was substantially higher in the anti‐inflammatory group (61 ± 13 vs. 38 ± 9 mg/dL, *p* < 0.001). Apolipoprotein measures further supported these findings, with higher ApoA1 (142 ± 21 vs. 105 ± 19 mg/dL, *p* < 0.001) and markedly lower ApoB (78 ± 16 vs. 129 ± 26 mg/dL, *p* < 0.001) levels among adherents. These findings show that adherence to an anti‐inflammatory diet improves lipid metabolism, reducing atherogenic lipids and increasing cardioprotective markers. The current data indicate that an anti‐inflammatory diet improves lipid metabolism, which may lower systemic inflammation and cancer‐promoting pathways. Elevated LDL‐C, triglycerides, and ApoB are associated with chronic inflammation, oxidative stress, and colorectal carcinogenesis via altered bile acid metabolism and endothelial dysfunction. The anti‐inflammatory group had higher HDL‐C and ApoA1 levels, which facilitate reverse cholesterol transport and inhibit inflammatory signaling (Roberts et al. 2006; Ungaro et al. 2020). Mediterranean diet adherence reduced LDL‐C and triglycerides while raising HDL‐C in patients with metabolic syndrome, and the PREDIMED trial found that a Mediterranean diet rich in olive oil or nuts improved lipid profiles and reduced cardiovascular events, which are colorectal cancer risk modifiers. In a recent study, higher DII scores were associated with poorer lipid profiles and CRC risk, supporting our finding that lower DII scores are associated with metabolic and oncologic outcomes (Estruch et al. 2018; Shivappa et al. 2018). An important finding from a large European cohort is that ApoB‐rich lipoproteins may increase carcinogenesis through lipid peroxidation and inflammatory signaling (Trites et al. 2023). These findings show that anti‐inflammatory diets improve traditional lipid markers and alter apolipoproteins, improving metabolic health and preventing colorectal cancer.

### 3.5 CBC Differences Between Anti‐Inflammatory Diet Adherents and Non‐Adherents

**FIGURE 2:**
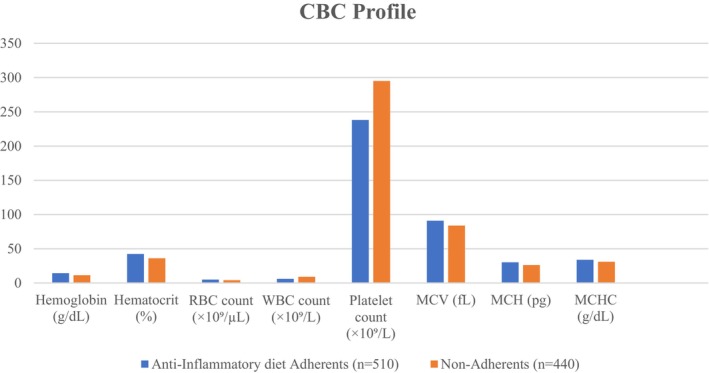
CBC differences between anti‐inflammatory diet adherents and non‐adherents.

Participants adhering to an anti‐inflammatory diet exhibited significantly more favorable hematological profiles compared to non‐adherents. Mean hemoglobin levels were higher in the adherent group (14.3 ± 1.0 vs. 11.3 ± 1.6 g/dL, *p* < 0.001), accompanied by increased hematocrit (42.4% ± 3.3% vs. 36.1% ± 4.4%, *p* < 0.001) and red blood cell counts (4.9 ± 0.4 vs. 4.1 ± 0.6 × 10^6^/μL, *p* = 0.005). Red cell indices were also significantly improved, with higher mean corpuscular volume (90.9 ± 4.7 vs. 83.8 ± 6.4 fL, *p* < 0.001), mean corpuscular hemoglobin (30.2 ± 2.0 vs. 26.2 ± 2.7 pg, *p* < 0.001), and mean corpuscular hemoglobin concentration (33.9 ± 1.1 vs. 30.9 ± 1.8 g/dL, *p* = 0.009). Conversely, inflammatory and stress‐related markers were elevated in the non‐adherent group, reflected by higher white blood cell counts (9.0 ± 2.0 vs. 6.0 ± 1.2 × 10^9^/L, *p* < 0.001) and platelet counts (295 ± 54 vs. 238 ± 39 × 10^9^/L, *p* = 0.004) (Figure 2). Low‐inflammatory diets may boost oxygen uptake, erythrocyte quality, and systemic inflammation. Dietary inflammation affects hematopoiesis and systemic immune modulation, as shown by hematological variations between adherents and non‐adherent high hemoglobin, hematocrit, and red cell indices indicate improved iron, folate, and vitamin B12 intake, which are needed for erythropoiesis but often deficient in pro‐inflammatory diets high in processed, nutrient‐poor foods non‐adherents have low hemoglobin and hematocrit, indicating chronic inflammatory anemia, where cytokines inhibit iron metabolism and red blood cell formation (Bracquez 2024; Wafula et al. 2021). The non‐adherent group's elevated WBC and platelet count supports the link between refined carbohydrates, saturated fats, and processed meats and leukocyte and platelet activation, chronic low‐grade inflammation, and tumor‐promoting microenvironments. This is supported by prospective studies like the PREDIMED trial, which found that a Mediterranean diet reduced systemic inflammation markers like leukocytes and platelets, and large cohort analyses that linked higher DII scores to lower hemoglobin and higher WBC counts (Aran et al. 2016). Adherents' improved red cell indices (MCV, MCH, and MCHC) support the function of nutrient‐rich anti‐inflammatory meals in maintaining red blood cell integrity and reducing microcytic or hypochromic alterations caused by iron deficiency and inflammatory anemia (Noreen et al. 2020). Chronic anemia, leukocytosis, and thrombocytosis are linked to poorer CRC prognosis; hence, these hematological benefits may indirectly prevent colorectal cancer. Overall, an anti‐inflammatory diet improves hematological health, lowers systemic inflammation, and may protect against cardiovascular disease and colorectal cancer. Previous studies have demonstrated that dietary patterns influence hematological indices, in part, through gut microbiota‐derived metabolites and inflammatory signaling, and that colorectal disease treatments, such as antibiotics or chemotherapy, can further modify leukocyte and platelet profiles via microbiome disruption.

### 3.6 Liver Enzymes Differences Between Anti‐Inflammatory Diet Adherents and Non‐Adherents

Participants adhering to an anti‐inflammatory diet demonstrated markedly better liver function profiles compared with non‐adherents. Liver enzyme activities were significantly lower in the adherent group including alanine aminotransferase (ALT: 23 ± 7 vs. 47 ± 16 U/L, *p* < 0.001), aspartate aminotransferase (AST: 20 ± 6 vs. 41 ± 14 U/L, *p* < 0.001), alkaline phosphatase (ALP: 82 ± 19 vs. 132 ± 37 U/L, *p* < 0.001), and γ‐glutamyl transferase (GGT: 23 ± 9 vs. 51 ± 18 U/L, *p* < 0.001). Bilirubin levels were also significantly reduced, with lower total bilirubin (0.7 ± 0.2 vs. 1.4 ± 0.4 mg/dL, *p* = 0.003) and direct bilirubin (0.17 ± 0.07 vs. 0.45 ± 0.13 mg/dL, *p* = 0.012) among adherents. Moreover, nutritional protein markers reflected a healthier profile in the anti‐inflammatory group, characterized by higher albumin concentrations (4.9 ± 0.3 vs. 3.7 ± 0.5 g/dL, *p* < 0.001), lower globulin levels (2.6 ± 0.3 vs. 3.7 ± 0.6 g/dL, *p* < 0.001), and a more favorable albumin‐to‐globulin ratio (1.9 ± 0.3 vs. 1.0 ± 0.2, *p* = 0.001) (Table 4). These data indicate that an anti‐inflammatory diet preserves hepatocellular integrity, reduces cholestatic stress, and boosts protein synthesis. The differences in liver enzymes and serum proteins between adherent and non‐adherent groups imply that anti‐inflammatory diets may protect the liver. High ALT, AST, ALP, and GGT levels in the non‐adherent group indicate hepatic stress, cholestasis, or NAFLD, which are connected to refined carbs, saturated fats, and processed meals (Amer 2018). Metabolism and hepatic inflammation are improved by lower liver enzyme levels. A higher A/G ratio suggests better hepatic production and less systemic inflammation in followers with higher albumin and lower globulin. Chronic low‐grade inflammation from pro‐inflammatory foods increases globulin fractions and suppresses albumin production, lowering protein quality (Bouhafs et al. 2024; Fic and Polak‐Szczybyło 2025; Kenđel Jovanović et al. 2021). Clinical and observational research like PREDIMED reveal that Mediterranean‐ and plant‐based anti‐inflammatory diets reduce liver fat, regulate transaminases, and improve protein profiles. Anti‐inflammatory diets reduce oxidative stress and hepatic lipid accumulation by providing polyphenols, omega‐3 fatty acids, fiber, and antioxidants, which boost protein synthesis and reduce hepatocellular damage (Fic and Polak‐Szczybyło 2025; Yang et al. 2020). Hepatoprotection may indirectly cut colorectal cancer risk by reducing systemic inflammation, insulin resistance, and bile acid dysregulation, which are major carcinogenesis pathways. An anti‐inflammatory diet improves liver enzyme profiles, serum protein balance, and hepatic inflammation, improving metabolic status and perhaps lowering colon cancer risk. Existing evidence suggests that diet‐induced alterations in the gut microbiota affect hepatic inflammation and enzyme activity through the gut–liver axis, whereas therapeutic interventions for intestinal diseases may exacerbate or ameliorate these effects depending on the dietary background.

**TABLE 4:** Liver enzymes differences between anti‐inflammatory diet adherents and non‐adherents.

Parameters	Anti‐inflammatory diet adherents (*n* = 515)	Non‐adherent (*n* = 435)	*p*
ALT (U/L)	23 ± 7	47 ± 16	< 0.001
AST (U/L)	20 ± 6	41 ± 14	< 0.001
ALP (U/L)	82 ± 19	132 ± 37	< 0.001
GGT (U/L)	23 ± 9	51 ± 18	< 0.001
Total bilirubin (mg/dL)	0.7 ± 0.2	1.4 ± 0.4	0.003
Direct bilirubin (mg/dL)	0.17 ± 0.07	0.45 ± 0.13	0.012
Albumin (g/dL)	4.9 ± 0.3	3.7 ± 0.5	< 0.001
Globulin (g/dL)	2.6 ± 0.3	3.7 ± 0.6	< 0.001
A/G ratio	1.9 ± 0.3	1.0 ± 0.2	0.001

*Note:* Values are presented as mean ± standard deviation (SD) for continuous variables and as percentages for categorical variables. Independent‐sample *t*‐tests were used to compare continuous variables.

### 3.7 Colorectal Cancer‐Related Biomarkers Differences Between Anti‐Inflammatory Diet Adherents and Non‐Adherents

Table 5 demonstrates pronounced differences in colorectal cancer‐related biomarkers between anti‐inflammatory diet adherents and non‐adherents. Systemic and local inflammatory markers were markedly lower among adherents, including CRP (1.5 ± 0.4 vs. 6.8 ± 1.8 mg/L, *p* < 0.001), IL‐6 (2.4 ± 0.8 vs. 9.2 ± 2.6 pg/mL, *p* < 0.001), TNF‐α (6.9 ± 1.9 vs. 17.4 ± 3.8 pg/mL, *p* < 0.001), and fecal calprotectin (68 ± 19 vs. 245 ± 75 μg/g, *p* < 0.001), whereas the anti‐inflammatory cytokine IL‐10 was significantly higher (13.1 ± 3.1 vs. 4.2 ± 1.1 pg/mL, *p* = 0.002). Tumor‐associated biomarkers were also substantially reduced in adherents including CEA (1.9 ± 0.5 vs. 7.9 ± 2.3 ng/mL, *p* < 0.001), MMP‐9 (94 ± 27 vs. 289 ± 88 ng/mL, *p* < 0.001), COX‐2 expression (0.5 ± 0.2 vs. 2.6 ± 0.6, *p* < 0.001), VEGF (106 ± 30 vs. 266 ± 62 pg/mL, *p* < 0.001), and NF‐κB activity (0.55 ± 0.11 vs. 1.31 ± 0.25 AU, *p* = 0.004). Growth and proliferative markers further highlighted protective effects of the anti‐inflammatory diet, with higher IGF‐1 (206 ± 35 vs. 134 ± 29 ng/mL, *p* = 0.007), lower Ki‐67 proliferation index (11% ± 3% vs. 38% ± 9%, *p* < 0.001), and reduced β‐catenin nuclear expression (10% vs. 41%, *p* < 0.001). Adherents had lower rates of genes linked to colorectal cancer such as p53 mutations (7% vs. 36%, *p* < 0.001), microsatellite instability (5% vs. 26%, *p* < 0.001), BRAF mutations (3% vs. 18%, *p* = 0.014), and APC mutations (6% vs. 29%, p < 0.001). An anti‐inflammatory diet is highly linked to decreased rates of genetic instability, oncogenic mutations, and pro‐inflammatory, pro‐angiogenic, and tumor‐promoting biomarkers. The disparities in CRC biomarkers between adherents and non‐adherents show how food affects colorectal carcinogenesis. Increased CRP, IL‐6, TNF‐α, and fecal calprotectin in non‐adherents indicate persistent systemic and gut‐specific inflammation, known to induce colorectal tumor development and progression (Florescu et al. 2023). In contrast, adherents with more IL‐10 have more anti‐inflammatory cytokines, which may offset the pro‐tumorigenic milieu. Anti‐inflammatory diet devotees had considerably lower CEA, MMP‐9, COX‐2, and VEGF indicators (Koper‐Lenkiewicz et al. 2021). COX‐2 overexpression and VEGF‐mediated angiogenesis are critical in early tumor growth and metastasis, whereas MMP‐9 facilitates extracellular matrix degradation and invasion. Lower levels of these markers in adherents align with prior studies showing that diets rich in fruits, vegetables, omega‐3 fatty acids, and polyphenols reduce COX‐2 activity, inhibit MMP expression, and attenuate VEGF‐mediated angiogenesis (Luo and Chen 2024). NF‐κB, a central transcription factor in inflammation‐driven carcinogenesis, was also suppressed in the anti‐inflammatory group, consistent with evidence that plant‐based diets and omega‐3 fatty acids inhibit NF‐κB signaling, thereby reducing inflammatory cytokine transcription and tumor‐promoting processes (Malakar 2020). IGF‐1 levels were higher in adherents, which may reflect better nutritional status and anabolic balance, although IGF‐1 can be context‐dependent in CRC risk. Genetic and proliferative markers demonstrated pronounced protection among adherents. Frequencies of p53, APC, and BRAF mutations, as well as β‐catenin nuclear translocation and Ki‐67 proliferation index, were all significantly lower. These alterations are known contributors to colorectal carcinogenesis through disruption of cell‐cycle control, Wnt signaling, and apoptosis (da Silva Barros et al. 2021). The lower prevalence of microsatellite instability in adherents further suggests reduced genomic instability and mutational burden, consistent with studies linking anti‐inflammatory dietary patterns to improved DNA repair and reduced oxidative DNA damage (Wang et al. 2023). An integrated molecular route combining systemic inflammation, tumor‐promoting signaling, and genomic stability shows anti‐inflammatory diets may reduce CRC risk. Consistent with these findings, previous studies have demonstrated that gut microbiome profiles can be integrated with dietary risk factors to develop diagnostic and risk‐prediction models for colorectal cancer, highlighting the clinical relevance of diet–microbiome interactions in CRC pathogenesis (Yang et al. 2019a). These findings are consistent with observational evidence indicating that dietary patterns low in refined carbohydrates and processed meats and enriched in fiber, polyphenols, and omega‐3 fatty acids are associated with reduced systemic inflammation, attenuation of tumor growth, and favorable modulation of colorectal cancer–preventive molecular biomarkers. Although somatic mutational burden was not directly assessed in the present study, accumulating evidence suggests that diet may indirectly influence genomic instability through microbiome‐mediated inflammatory signaling, oxidative stress, and microbial genotoxin production, thereby promoting DNA damage and mutational accumulation in colorectal epithelial cells.

**TABLE 5:** Colorectal cancer‐related biomarkers differences between anti‐inflammatory diet adherents and non‐adherents.

Biomarkers	Anti‐inflammatory diet adherents (*n* = 515)	Non‐adherent (*n* = 435)	*p*
CRP (mg/L)	1.5 ± 0.4	6.8 ± 1.8	< 0.001
IL‐6 (pg/mL)	2.4 ± 0.8	9.2 ± 2.6	< 0.001
TNF‐α (pg/mL)	6.9 ± 1.9	17.4 ± 3.8	< 0.001
IL‐10 (pg/mL)	13.1 ± 3.1	4.2 ± 1.1	0.002
Fecal calprotectin (μg/g)	68 ± 19	245 ± 75	< 0.001
CEA (ng/mL)	1.9 ± 0.5	7.9 ± 2.3	< 0.001
MMP‐9 (ng/mL)	94 ± 27	289 ± 88	< 0.001
COX‐2 expression (0–3)	0.5 ± 0.2	2.6 ± 0.6	< 0.001
VEGF (pg/mL)	106 ± 30	266 ± 62	< 0.001
NF‐κB activity (AU)	0.55 ± 0.11	1.31 ± 0.25	0.004
IGF‐1 (ng/mL)	206 ± 35	134 ± 29	0.007
p53 mutation frequency (%)	7%	36%	< 0.001*
Ki‐67 proliferation index (%)	11 ± 3	38 ± 9	< 0.001
Microsatellite instability (%)	5%	26%	< 0.001*
BRAF mutation (%)	3%	18%	0.014*
APC mutation (%)	6%	29%	< 0.001*
β‐catenin nuclear expression (%)	10%	41%	< 0.001*

*Note:* Values are presented as mean ± standard deviation (SD) for continuous variables and as percentages for categorical variables. Independent‐sample *t*‐tests were used to compare continuous variables, and Chi‐square tests (*) were applied for categorical outcomes.

### 3.8 Bacterial Species Differences Between Anti‐Inflammatory Diet Adherents and Non‐Adherents

Participants adhering to an anti‐inflammatory diet demonstrated a significantly healthier gut microbiota composition compared with non‐adherents. Beneficial bacterial genera were more abundant among adherents, including *Bifidobacterium* (4.5 ± 1.2 × 10^8^ vs. 3.2 ± 1.0 × 10^8^ CFU/mL, *p* < 0.001), *Lactobacillus* (3.8 ± 0.9 × 10^8^ vs. 2.7 ± 0.8 × 10^8^ CFU/mL, *p* < 0.001), *A. muciniphila* (1.7 ± 0.5 × 10^8^ vs. 1.1 ± 0.4 × 10^8^ CFU/mL, *p* < 0.001), *F. prausnitzii* (2.1 ± 0.6 × 10^8^ vs. 1.3 ± 0.5 × 10^8^ CFU/mL, *p* < 0.001), and *Roseburia* spp. (1.9 ± 0.5 × 10^8^ vs. 1.1 ± 0.4 × 10^8^ CFU/mL, *p* < 0.001), all of which are short‐chain fatty acid (SCFA) producers linked to intestinal barrier protection and anti‐inflammatory effects. Conversely, pathogenic and pro‐carcinogenic species were significantly reduced among adherents including *F. nucleatum* (1.1 ± 0.4 × 10^8^ vs. 2.3 ± 0.7 × 10^8^ CFU/mL, *p* < 0.001), pathogenic strains of *E. coli* (0.9 ± 0.3 × 10^8^ vs. 1.8 ± 0.5 × 10^8^ CFU/mL, *p* < 0.001), and *C. difficile* (0.5 ± 0.2 × 10^8^ vs. 1.2 ± 0.4 × 10^8^ CFU/mL, *p* < 0.001) (Table 6). These findings support prior research indicating fiber, polyphenols, and unsaturated fats increase SCFA‐producing bacteria. For instance, a large‐scale metagenomic study showed link plant‐based, high‐fiber diets with enrichment of *Bifidobacterium* and *Lactobacillus* (Jagelavičiūtė et al. 2025). It was also demonstrated that adherence to a Mediterranean‐style diet increased *Akkermansia* and *Faecalibacterium* while reducing inflammatory microbes (Khavandegar et al. 2024). Similarly, it was also reported that overrepresentation of *F. nucleatum* in colorectal cancer tissues, supporting its role as a microbial biomarker of tumorigenesis (Wang and Fang 2023). The reduction of pathogenic *E. coli* and *C. difficile* in Anti‐Inflammatory adherents aligns with prior evidence that high‐fiber, anti‐inflammatory diets improve microbial diversity and limit the proliferation of opportunistic pathogens (Wu et al. 2022). Collectively, these results reinforce the role of an anti‐inflammatory diet in promoting a eubiotic gut environment that protects against the development of colorectal cancer through microbial modulation. Prior study report that both habitual diet and therapeutic interventions for intestinal inflammatory diseases significantly alter microbial composition across multiple biological compartments, including stool, saliva, serum, and urine, as demonstrated in patients receiving anti‐TNF‐α therapy (Park et al. 2022), supporting the relevance of diet–microbiome interactions observed in the present study.

**TABLE 6:** Bacterial species differences between anti‐inflammatory diet adherents and non‐adherents.

Bacterial species	Anti‐inflammatory diet adherents (*n* = 515, CFU/mL)	Non‐adherent (*n* = 435, CFU/mL)	*p*
*Bifidobacterium*	4.5 ± 1.2 × 10^8^	3.2 ± 1.0 × 10^8^	< 0.001
*Lactobacillus*	3.8 ± 0.9 × 10^8^	2.7 ± 0.8 × 10^8^	< 0.001
*Akkermansia muciniphila*	1.7 ± 0.5 × 10^8^	1.1 ± 0.4 × 10^8^	< 0.001
*Faecalibacterium prausnitzii*	2.1 ± 0.6 × 10^8^	1.3 ± 0.5 × 10^8^	< 0.001
*Roseburia* spp.	1.9 ± 0.5 × 10^8^	1.1 ± 0.4 × 10^8^	< 0.001
*Fusobacterium nucleatum*	1.1 ± 0.4 × 10^8^	2.3 ± 0.7 × 10^8^	< 0.001
*Escherichia coli* (pathogenic strains)	0.9 ± 0.3 × 10^8^	1.8 ± 0.5 × 10^8^	< 0.001
*Clostridium difficile*	0.5 ± 0.2 × 10^8^	1.2 ± 0.4 × 10^8^	< 0.001

*Note:* Values are illustrative mean ± SD (CFU/mL). *p*‐values derived from independent‐sample *t*‐tests.

### 3.9 Multiple Logistic Regression Analysis Showing Association Between Anti‐Inflammatory Diet Adherence and Clinical, Biomarker, and Microbiota Outcomes in Colorectal Cancer Development

Multiple logistic regression analysis showed that CRC patients who followed an anti‐inflammatory diet had better clinical, biochemical, and microbiome outcomes (Table 7). After controlling for age, sex, BMI, smoking status, and total energy intake, the anti‐inflammatory diet was associated with a significantly lower risk of obesity (*β* = −1.90, SE = 0.26, OR = 0.15, 95% CI: 0.09–0.25, *p* < 0.001) and central obesity (*β* = −2.12, SE = 0.27, OR = 0.12, 95% CI: 0.07–0.20, *p* < 0.001). Similarly, dyslipidemia, anemia, leukocytosis, and increased liver enzymes had reduced risks (*β* = −1.77, SE = 0.25, OR = 0.17, 0.26, 0.19, 0.14, 0.27, and 0.16; all *p* < 0.001). Anti‐inflammatory diet adherence significantly reduced systemic and cytokine‐mediated inflammation (*p* < 0.001) by inversely affecting CRP (*β* = −2.30, SE = 0.27, OR = 0.10, 95% CI: 0.06–0.17) and IL‐6 levels (*β* = −2.21, SE = 0.26, OR = 0.11, 95% CI: 0.07–0.18). Adherent individuals had significantly reduced levels of tumor markers such as CEA (*β* = −2.41, SE = 0.28, OR = 0.09) and MMP‐9 (*β* = −2.12, SE = 0.27, OR = 0.12; both *p* < 0.001). Adherence was linked to lower odds of p53 mutation, Ki‐67 overexpression, microsatellite instability, APC mutation, and β‐catenin nuclear expression (*p* < 0.001). The gut microbiota analysis showed a higher prevalence of beneficial bacteria (*Bifidobacterium*, *Lactobacillu*s, *F. prausnitzii* , and *A. muciniphila* ) and lower prevalence of pathogenic species ( *F. nucleatum* , *C. difficile* ) (*β* = 0.79, OR = 2.20, OR = 0.74, OR = 2.10, *β* = 0.83). These data suggest anti‐inflammatory diets improve gut microbiota and lower colon cancer risk. Statistics show that anti‐inflammatory diets prevent various types of colorectal cancer. Reducing obesity, central adiposity, and dyslipidemia improves diet‐induced systemic inflammation and insulin sensitivity, which affect colorectal carcinogenesis (Shahinfar et al. 2022). Increased micronutrient intake and lower chronic inflammatory burden reduce anemia and leukocytosis, supporting anti‐inflammatory diets' effects on inflammatory cytokines and erythropoiesis. Low liver enzymes and systemic inflammatory biomarkers (CRP, IL‐6) imply anti‐inflammatory diet followers minimize metabolic and hepatic stress, which causes colon cancer (da Silva Barros et al. 2021; Li et al. 2022). The adherent group showed significant decreases in tumor‐related biomarkers and genetic alterations such as CEA, MMP‐9, p53, APC, Ki‐67, MSI, and β‐catenin. Systemic inflammation, colonic microenvironment, cellular proliferation, and genetic stability can be affected by diet. Previous research suggests that diets high in fiber, polyphenols, and omega‐3 fatty acids inhibit COX‐2 expression, NF‐κB activity, and colonic tissue proliferation, reducing CRC incidence, and progression (Estruch et al. 2018; Padmanabhan et al. 2018). Data suggests anti‐inflammatory diets greatly reduce colon cancer risk. It improves gut microbiota and reduces metabolic, inflammatory, and genetic changes that cause tumors. By increasing good bacteria and decreasing bad bacteria, food protects gut and systemic cancer. Interventional studies using synbiotic formulations further support the capacity of diet to reshape microbial communities and their functional outputs, which may underlie the associations observed in the present regression models.

**TABLE 7:** Multiple logistic regression analysis showing association between anti‐inflammatory diet adherence and clinical, biomarker, and microbiota outcomes in colorectal cancer development.

Parameter category (*n* = 950)	Key outcome	*β* (coefficient)	SE	Adjusted OR (95% CI)	*p*	Interpretation
Anthropometric	Obesity (BMI ≥ 30 kg/m^2^)	−1.90	0.26	0.15 (0.09–0.25)	< 0.001	85% ↓ risk of obesity
	Central obesity (%)	−2.12	0.27	0.12 (0.07–0.20)	< 0.001	88% ↓ risk
Lipid profile	Dyslipidemia (%)	−1.77	0.25	0.17 (0.10–0.27)	< 0.001	83% ↓ risk
CBC (hematology)	Anemia prevalence (%)	−1.66	0.26	0.19 (0.11–0.31)	< 0.001	81% ↓ risk
	Leukocytosis (WBC > 10 × 10^9^/L)	−1.97	0.29	0.14 (0.08–0.25)	< 0.001	86% ↓ risk
Liver function	Elevated ALT/AST (%)	−1.83	0.27	0.16 (0.09–0.28)	< 0.001	84% ↓ risk
Inflammatory biomarkers	High CRP (> 5 mg/L)	−2.30	0.27	0.10 (0.06–0.17)	< 0.001	90% ↓ systemic inflammation
	Elevated IL‐6 (> 7 pg/mL)	−2.21	0.26	0.11 (0.07–0.18)	< 0.001	89% ↓ IL‐6
Tumor markers	Elevated CEA (> 5 ng/mL)	−2.41	0.28	0.09 (0.05–0.16)	< 0.001	91% ↓ tumor marker elevation
	High MMP‐9 (> 250 ng/mL)	−2.12	0.27	0.12 (0.07–0.21)	< 0.001	88% ↓ ECM degradation
Genetic/molecular markers	p53 mutation (%)	−2.04	0.27	0.13 (0.08–0.23)	< 0.001	87% ↓ mutation likelihood
	Ki‐67 high (> 25%)	−2.21	0.26	0.11 (0.07–0.18)	< 0.001	89% ↓ cell proliferation
	Microsatellite instability (MSI)	−1.90	0.29	0.15 (0.08–0.27)	< 0.001	85% ↓ genomic instability
	APC mutation (%)	−1.97	0.27	0.14 (0.08–0.25)	< 0.001	86% ↓ risk
	β‐catenin nuclear expression (%)	−1.71	0.28	0.18 (0.10–0.31)	< 0.001	82% ↓ Wnt activation
Gut microbiota	*Bifidobacterium* ↑	0.79	0.21	2.20 (1.45–3.33)	< 0.001	↑ beneficial species
	*Lactobacillus* ↑	0.74	0.21	2.10 (1.38–3.19)	< 0.001	↑ beneficial species
	*Faecalibacterium prausnitzii* ↑	0.83	0.23	2.30 (1.47–3.61)	< 0.001	↑ anti‐inflammatory species
	*Akkermansia muciniphila* ↑	0.81	0.24	2.25 (1.40–3.60)	< 0.001	↑ mucosal protective species
	*Fusobacterium nucleatum* ↓	−1.71	0.27	0.18 (0.11–0.31)	< 0.001	82% ↓ pathogenic species
	*Clostridium difficile* ↓	−1.77	0.27	0.17 (0.10–0.29)	< 0.001	83% ↓ pathogenic species
	Pathogenic *Escherichia coli* ↓	−1.83	0.27	0.16 (0.09–0.28)	< 0.001	84% ↓ pathogenic species

*Note:* Values represent adjusted odds ratios (ORs) with 95% confidence intervals (CIs) derived from multivariate logistic regression analyses, controlling for age, sex, BMI, smoking status, physical activity, and total caloric intake.

Abbreviations: *β*, standardized regression coefficient; SE, standard error.

## 4 Conclusion

This study demonstrates that adherence to an anti‐inflammatory diet significantly reduces risk factors associated with colorectal cancer by improving anthropometric, biochemical, inflammatory, genetic, and microbial profiles. Individuals following this dietary pattern exhibited lower prevalence of obesity, dyslipidemia, anemia, and liver dysfunction, along with reduced systemic inflammation, tumor biomarkers, and oncogenic mutations. At the same time, beneficial gut microbiota were enriched while pathogenic species were suppressed, indicating a dual protective effect at both systemic and intestinal levels. This study suggests that anti‐inflammatory diets may prevent colorectal cancer and promote health cost‐effectively and noninvasively. Broad‐scale longitudinal and interventional research is needed to prove causality. Multi‐omics techniques may illuminate diet, genes, and microorganisms, leading to colorectal cancer prevention nutrition.

## 5 Limitations of the Study

Given the retrospective observational design, causal inferences cannot be drawn between food patterns, gut dysbiosis, inflammatory biomarkers, and colorectal cancer; reverse causation is also possible. The relationships may have been affected by residual confounding from unmeasured lifestyle or clinical factors, and by the lack of direct assessment of somatic mutational burden.

## Author Contributions

Hantao Wang and Yunjie Shi contributed to the conception and design of the study and drafted the initial manuscript. Wei Wang and Xu Li assisted in data acquisition, patient recruitment, and clinical coordination.

## Funding

The authors have nothing to report.

## Ethics Statement

This study was conducted in accordance with the principles of the Declaration of Helsinki and applicable national and institutional guidelines. Ethics approval was obtained from the Ethics Committee of Shanghai Changle Hospital, China, approval no. SC26111231.

## Data Availability

The data that support the findings of this study are available on request from the corresponding author. The data are not publicly available due to privacy or ethical restrictions.
